# Methyl 4-but­oxy-3-methoxy­benzoate

**DOI:** 10.1107/S1600536809003080

**Published:** 2009-01-31

**Authors:** Min Zhang, Ran-Zhe Lu, Lu-Na Han, Wen-Bin Wei, Hai-Bo Wang

**Affiliations:** a College of Science, Nanjing University of Technology, Xinmofan Road No. 5 Nanjing, Nanjing 210009, People’s Republic of China

## Abstract

The title compound, C_13_H_18_O_4_, is an inter­mediate product in the synthesis of quinazoline derivatives. Crystal structure analysis shows that the benzene–butoxy C_ar_—O—C—C torsion angle is 175.3 (2)° and that the benzene–methoxycarbonyl C_ar_—C—O—C torsion angle is 175.2 (2)°. Torsion angles close to 180° indicate that the molecule is almost planar.

## Related literature

For general background, see: Knesl *et al.* (2006 ▶). For bond-length data, see: Allen *et al.* (1987 ▶).
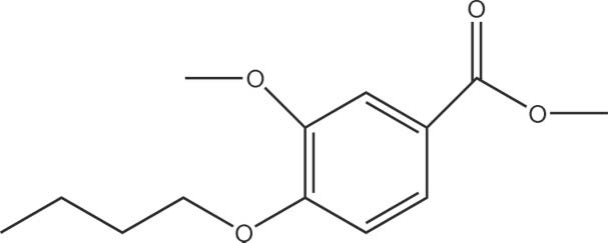


## Experimental

### 

#### Crystal data

C_13_H_18_O_4_
*M* *_r_* = 238.27
Triclinic, 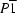

*a* = 7.9660 (16) Å
*b* = 9.1630 (18) Å
*c* = 10.143 (2) Å
α = 64.80 (2)°
β = 70.96 (3)°
γ = 79.26 (3)°
*V* = 632.3 (2) Å^3^
*Z* = 2
Mo *K*α radiation
μ = 0.09 mm^−1^
*T* = 293 (2) K
0.30 × 0.20 × 0.10 mm

#### Data collection

Enraf–Nonius CAD-4 diffractometer
Absorption correction: ψ scan (North *et al.*, 1968 ▶) *T*_min_ = 0.973, *T*_max_ = 0.991
2474 measured reflections
2294 independent reflections
1567 reflections with *I* > 2σ(*I*)
*R*_int_ = 0.067
3 standard reflections every 200 reflections intensity decay: 1%

#### Refinement

*R*[*F* ^2^ > 2σ(*F* ^2^)] = 0.064
*wR*(*F* ^2^) = 0.168
*S* = 1.00
2294 reflections
154 parameters
H-atom parameters constrained
Δρ_max_ = 0.18 e Å^−3^
Δρ_min_ = −0.21 e Å^−3^

### 

Data collection: *CAD-4 EXPRESS* (Enraf–Nonius, 1994 ▶); cell refinement: *CAD-4 EXPRESS*; data reduction: *XCAD4* (Harms & Wocadlo, 1995 ▶); program(s) used to solve structure: *SHELXS97* (Sheldrick, 2008 ▶); program(s) used to refine structure: *SHELXL97* (Sheldrick, 2008 ▶); molecular graphics: *SHELXTL* (Sheldrick, 2008 ▶); software used to prepare material for publication: *PLATON* (Spek, 2003 ▶).

## Supplementary Material

Crystal structure: contains datablocks global, I. DOI: 10.1107/S1600536809003080/wk2098sup1.cif

Structure factors: contains datablocks I. DOI: 10.1107/S1600536809003080/wk2098Isup2.hkl

Additional supplementary materials:  crystallographic information; 3D view; checkCIF report

## Figures and Tables

**Fig. 1.**
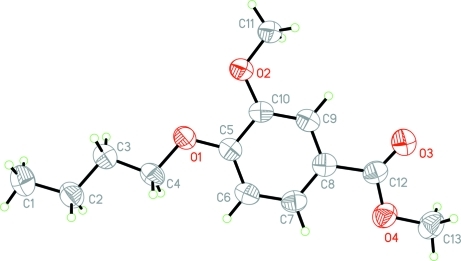
The molecular structure of the title molecule, with the atom-numbering scheme.

## supplementary crystallographic information

### Comment

As part of our ongoing studies on quinazoline derivatives (Knesl *et al.*, 2006), we report herein the crystal structure of the title compound.

In the molecule of the title compound (Fig 1), the bond lengths (Allen *et al.*,  1987) and angles are within normal ranges. Ring A (C4–C9) is, of course, planar.

### Experimental

For the preparation of the title compound, methyl 3-methoxy-4-hydroxybenzoate (55 mmol), 1-bromobutane (110 mmol) and potassium carbonate (165 mmol) were mixed with DMF (60 ml), and then the mixture was heated to reflux for 2 h. Reaction progress was monitored by TLC. After cooling and filtration, the title compound was obtained (yield 92%, m.p. 317 K). Crystals suitable for X-ray analysis were obtained by slow evaporation of an ethyl acetate solution.

### Refinement

H atoms were positioned geometrically, with C—H = 0.93, 0.97 and 0.96 Å for aromatic, methylene and methyl H, respectively, and constrained to ride on their parent atoms, with U_iso_(H) = xU_eq_(C), where x = 1.5 for methyl H and x = 1.2 for all other H atoms.

### Crystal data

**Table:**

C_13_H_18_O_4_	*Z* = 2
*M**_r_* = 238.27	*F*(000) = 256
Triclinic, *P*1	*D*_x_ = 1.252 Mg m^−^^3^
Hall symbol: -P 1	Melting point: 317 K
*a* = 7.9660 (16) Å	Mo *K*α radiation, λ = 0.71073 Å
*b* = 9.1630 (18) Å	Cell parameters from 25 reflections
*c* = 10.143 (2) Å	θ = 9–12°
α = 64.80 (2)°	µ = 0.09 mm^−^^1^
β = 70.96 (3)°	*T* = 293 K
γ = 79.26 (3)°	Block, colourless
*V* = 632.3 (2) Å^3^	0.30 × 0.20 × 0.10 mm

### Data collection

**Table:**

Enraf–Nonius CAD-4 diffractometer	1567 reflections with *I* > 2σ(*I*)
Radiation source: fine-focus sealed tube	*R*_int_ = 0.067
graphite	θ_max_ = 25.3°, θ_min_ = 2.3°
ω/2θ scans	*h* = 0→9
Absorption correction: ψ scan (North *et al.*, 1968)	*k* = −10→11
*T*_min_ = 0.973, *T*_max_ = 0.991	*l* = −11→12
2474 measured reflections	3 standard reflections every 200 reflections
2294 independent reflections	intensity decay: 1%

### Refinement

**Table:**

Refinement on *F*^2^	Primary atom site location: structure-invariant direct methods
Least-squares matrix: full	Secondary atom site location: difference Fourier map
*R*[*F*^2^ > 2σ(*F*^2^)] = 0.064	Hydrogen site location: inferred from neighbouring sites
*wR*(*F*^2^) = 0.168	H-atom parameters constrained
*S* = 1.00	*w* = 1/[σ^2^(*F*_o_^2^) + (0.07*P*)^2^ + 0.43*P*] where *P* = (*F*_o_^2^ + 2*F*_c_^2^)/3
2294 reflections	(Δ/σ)_max_ < 0.001
154 parameters	Δρ_max_ = 0.18 e Å^−^^3^
0 restraints	Δρ_min_ = −0.21 e Å^−^^3^

### Special details

**Table:**

Geometry. All esds (except the esd in the dihedral angle between two l.s. planes) are estimated using the full covariance matrix. The cell esds are taken into account individually in the estimation of esds in distances, angles and torsion angles; correlations between esds in cell parameters are only used when they are defined by crystal symmetry. An approximate (isotropic) treatment of cell esds is used for estimating esds involving l.s. planes.
Refinement. Refinement of F^2^ against ALL reflections. The weighted R-factor wR and goodness of fit S are based on F^2^, conventional R-factors R are based on F, with F set to zero for negative F^2^. The threshold expression of F^2^ > 2sigma(F^2^) is used only for calculating R-factors(gt) etc. and is not relevant to the choice of reflections for refinement. R-factors based on F^2^ are statistically about twice as large as those based on F, and R- factors based on ALL data will be even larger.

### Fractional atomic coordinates and isotropic or equivalent isotropic displacement parameters (Å^2^)

**Table:**

	*x*	*y*	*z*	*U*_iso_*/*U*_eq_	
O1	0.3147 (2)	0.6241 (2)	0.05115 (19)	0.0488 (5)	
O2	0.0656 (2)	0.4271 (2)	0.15700 (19)	0.0516 (5)	
O3	0.0117 (3)	0.1304 (3)	0.7289 (2)	0.0733 (7)	
O4	0.1887 (2)	0.2708 (2)	0.75552 (19)	0.0570 (5)	
C1	0.6296 (5)	1.0667 (4)	−0.4050 (3)	0.0749 (10)	
H1A	0.7158	1.1438	−0.4358	0.112*	
H1B	0.5201	1.1226	−0.4245	0.112*	
H1C	0.6732	0.9990	−0.4612	0.112*	
C2	0.5977 (4)	0.9645 (4)	−0.2387 (3)	0.0606 (8)	
H2A	0.5569	1.0348	−0.1838	0.073*	
H2B	0.7101	0.9117	−0.2204	0.073*	
C3	0.4645 (3)	0.8369 (3)	−0.1746 (3)	0.0473 (6)	
H3A	0.3493	0.8884	−0.1865	0.057*	
H3B	0.5016	0.7675	−0.2305	0.057*	
C4	0.4487 (4)	0.7377 (3)	−0.0111 (3)	0.0493 (7)	
H4A	0.5619	0.6806	0.0002	0.059*	
H4B	0.4189	0.8078	0.0438	0.059*	
C5	0.2767 (3)	0.5331 (3)	0.2032 (3)	0.0410 (6)	
C6	0.3595 (3)	0.5399 (3)	0.3000 (3)	0.0495 (7)	
H6A	0.4504	0.6098	0.2622	0.059*	
C7	0.3085 (3)	0.4438 (3)	0.4524 (3)	0.0477 (6)	
H7A	0.3639	0.4505	0.5172	0.057*	
C8	0.1752 (3)	0.3373 (3)	0.5099 (3)	0.0443 (6)	
C9	0.0934 (3)	0.3285 (3)	0.4133 (3)	0.0438 (6)	
H9A	0.0051	0.2559	0.4516	0.053*	
C10	0.1400 (3)	0.4256 (3)	0.2606 (3)	0.0401 (6)	
C11	−0.0929 (4)	0.3439 (4)	0.2120 (3)	0.0564 (8)	
H11A	−0.1314	0.3542	0.1278	0.085*	
H11B	−0.1841	0.3895	0.2749	0.085*	
H11C	−0.0703	0.2317	0.2702	0.085*	
C12	0.1161 (3)	0.2332 (3)	0.6753 (3)	0.0468 (6)	
C13	0.1295 (4)	0.1866 (4)	0.9164 (3)	0.0661 (8)	
H13A	0.1890	0.2239	0.9638	0.099*	
H13B	0.1562	0.0729	0.9425	0.099*	
H13C	0.0034	0.2066	0.9511	0.099*	

### Atomic displacement parameters (Å^2^)

**Table:**

	*U*^11^	*U*^22^	*U*^33^	*U*^12^	*U*^13^	*U*^23^
O1	0.0478 (10)	0.0490 (11)	0.0479 (10)	−0.0110 (8)	−0.0085 (8)	−0.0176 (8)
O2	0.0480 (10)	0.0598 (12)	0.0497 (10)	−0.0140 (9)	−0.0114 (8)	−0.0211 (9)
O3	0.0858 (16)	0.0749 (15)	0.0559 (12)	−0.0289 (13)	−0.0188 (11)	−0.0135 (11)
O4	0.0592 (12)	0.0667 (13)	0.0460 (10)	−0.0054 (10)	−0.0141 (9)	−0.0226 (9)
C1	0.076 (2)	0.065 (2)	0.066 (2)	−0.0173 (18)	−0.0084 (16)	−0.0119 (16)
C2	0.0603 (18)	0.0566 (18)	0.0607 (17)	−0.0145 (15)	−0.0097 (14)	−0.0198 (14)
C3	0.0466 (15)	0.0402 (15)	0.0547 (15)	0.0003 (12)	−0.0074 (11)	−0.0241 (12)
C4	0.0486 (15)	0.0425 (15)	0.0589 (16)	−0.0092 (12)	−0.0099 (12)	−0.0228 (12)
C5	0.0390 (13)	0.0368 (13)	0.0485 (13)	0.0017 (11)	−0.0093 (10)	−0.0215 (11)
C6	0.0443 (15)	0.0510 (16)	0.0575 (16)	−0.0088 (12)	−0.0119 (12)	−0.0245 (13)
C7	0.0444 (15)	0.0497 (16)	0.0563 (15)	0.0033 (12)	−0.0201 (12)	−0.0254 (12)
C8	0.0394 (13)	0.0425 (14)	0.0528 (15)	0.0072 (11)	−0.0137 (11)	−0.0236 (12)
C9	0.0403 (14)	0.0397 (14)	0.0536 (15)	0.0013 (11)	−0.0115 (11)	−0.0228 (11)
C10	0.0361 (13)	0.0417 (14)	0.0498 (14)	0.0055 (11)	−0.0125 (10)	−0.0274 (11)
C11	0.0539 (17)	0.0673 (19)	0.0517 (15)	−0.0164 (15)	−0.0143 (12)	−0.0217 (14)
C12	0.0418 (14)	0.0487 (16)	0.0534 (15)	0.0075 (12)	−0.0184 (12)	−0.0235 (12)
C13	0.0680 (19)	0.079 (2)	0.0490 (16)	−0.0016 (17)	−0.0149 (14)	−0.0253 (15)

### Geometric parameters (Å, °)

**Table:**

O1—C5	1.364 (3)	C4—H4B	0.9700
O1—C4	1.427 (3)	C5—C6	1.375 (3)
O2—C10	1.359 (3)	C5—C10	1.411 (3)
O2—C11	1.423 (3)	C6—C7	1.376 (4)
O3—C12	1.198 (3)	C6—H6A	0.9300
O4—C12	1.317 (3)	C7—C8	1.385 (3)
O4—C13	1.429 (3)	C7—H7A	0.9300
C1—C2	1.501 (4)	C8—C9	1.376 (3)
C1—H1A	0.9600	C8—C12	1.495 (4)
C1—H1B	0.9600	C9—C10	1.379 (3)
C1—H1C	0.9600	C9—H9A	0.9300
C2—C3	1.510 (4)	C11—H11A	0.9600
C2—H2A	0.9700	C11—H11B	0.9600
C2—H2B	0.9700	C11—H11C	0.9600
C3—C4	1.488 (4)	C13—H13A	0.9600
C3—H3A	0.9700	C13—H13B	0.9600
C3—H3B	0.9700	C13—H13C	0.9600
C4—H4A	0.9700		
			
C5—O1—C4	116.99 (19)	C5—C6—C7	120.4 (2)
C10—O2—C11	117.84 (19)	C5—C6—H6A	119.8
C12—O4—C13	116.2 (2)	C7—C6—H6A	119.8
C2—C1—H1A	109.5	C6—C7—C8	120.5 (2)
C2—C1—H1B	109.5	C6—C7—H7A	119.8
H1A—C1—H1B	109.5	C8—C7—H7A	119.8
C2—C1—H1C	109.5	C9—C8—C7	119.4 (2)
H1A—C1—H1C	109.5	C9—C8—C12	119.2 (2)
H1B—C1—H1C	109.5	C7—C8—C12	121.4 (2)
C1—C2—C3	115.2 (3)	C8—C9—C10	121.2 (2)
C1—C2—H2A	108.5	C8—C9—H9A	119.4
C3—C2—H2A	108.5	C10—C9—H9A	119.4
C1—C2—H2B	108.5	O2—C10—C9	125.5 (2)
C3—C2—H2B	108.5	O2—C10—C5	115.7 (2)
H2A—C2—H2B	107.5	C9—C10—C5	118.9 (2)
C4—C3—C2	111.0 (2)	O2—C11—H11A	109.5
C4—C3—H3A	109.4	O2—C11—H11B	109.5
C2—C3—H3A	109.4	H11A—C11—H11B	109.5
C4—C3—H3B	109.4	O2—C11—H11C	109.5
C2—C3—H3B	109.4	H11A—C11—H11C	109.5
H3A—C3—H3B	108.0	H11B—C11—H11C	109.5
O1—C4—C3	110.7 (2)	O3—C12—O4	124.0 (2)
O1—C4—H4A	109.5	O3—C12—C8	123.9 (2)
C3—C4—H4A	109.5	O4—C12—C8	112.0 (2)
O1—C4—H4B	109.5	O4—C13—H13A	109.5
C3—C4—H4B	109.5	O4—C13—H13B	109.5
H4A—C4—H4B	108.1	H13A—C13—H13B	109.5
O1—C5—C6	125.3 (2)	O4—C13—H13C	109.5
O1—C5—C10	115.1 (2)	H13A—C13—H13C	109.5
C6—C5—C10	119.7 (2)	H13B—C13—H13C	109.5
			
C1—C2—C3—C4	177.4 (3)	C11—O2—C10—C5	−168.8 (2)
C5—O1—C4—C3	−175.3 (2)	C8—C9—C10—O2	−179.1 (2)
C2—C3—C4—O1	176.4 (2)	C8—C9—C10—C5	1.3 (4)
C4—O1—C5—C6	−1.4 (4)	O1—C5—C10—O2	0.2 (3)
C4—O1—C5—C10	178.3 (2)	C6—C5—C10—O2	179.9 (2)
O1—C5—C6—C7	179.0 (2)	O1—C5—C10—C9	179.8 (2)
C10—C5—C6—C7	−0.7 (4)	C6—C5—C10—C9	−0.4 (4)
C5—C6—C7—C8	1.0 (4)	C13—O4—C12—O3	−3.1 (4)
C6—C7—C8—C9	−0.2 (4)	C13—O4—C12—C8	175.2 (2)
C6—C7—C8—C12	−179.3 (2)	C9—C8—C12—O3	7.1 (4)
C7—C8—C9—C10	−0.9 (4)	C7—C8—C12—O3	−173.9 (3)
C12—C8—C9—C10	178.1 (2)	C9—C8—C12—O4	−171.2 (2)
C11—O2—C10—C9	11.6 (4)	C7—C8—C12—O4	7.8 (4)
